# Supplementary figures and images for: Pregnancy rest-activity patterns are related to salivary cortisol rhythms and maternal-fetal health indicators in women from a disadvantaged population
Source: PLoS One. 2020 Mar 3;15(3):e0229567. doi: 10.1371/journal.pone.0229567 (PMC7053712; doi:10.1371/journal.pone.0229567)

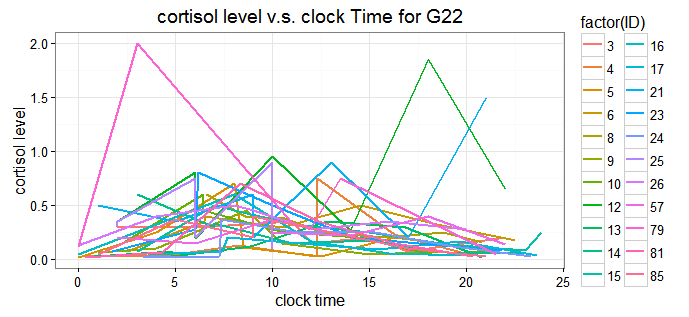


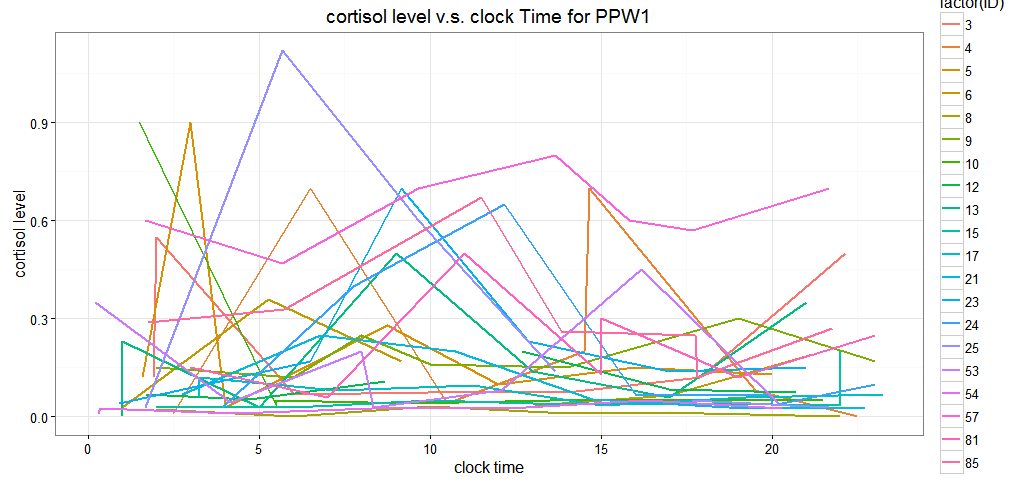

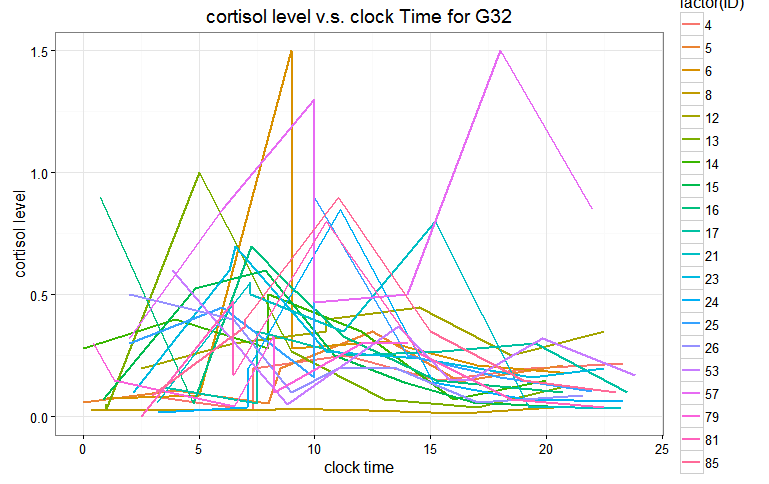

Supplement: S3 Fig — Spaghetti graphs of saliva cortisol concentration of each study participant during gestational week 22 (A), 32 (B) and postpartum week one (C). Cortisol level units are μg/dL. (DOCX) [file pone.0229567.s003.docx]
