# Supplementary material for: Pregnancy rest-activity patterns are related to salivary cortisol rhythms and maternal-fetal health indicators in women from a disadvantaged population
Source: PLoS One. 2020 Mar 3;15(3):e0229567. doi: 10.1371/journal.pone.0229567 (PMC7053712; doi:10.1371/journal.pone.0229567)
Supplement: Supplementary file 2 [file pone.0229567.s001.docx]

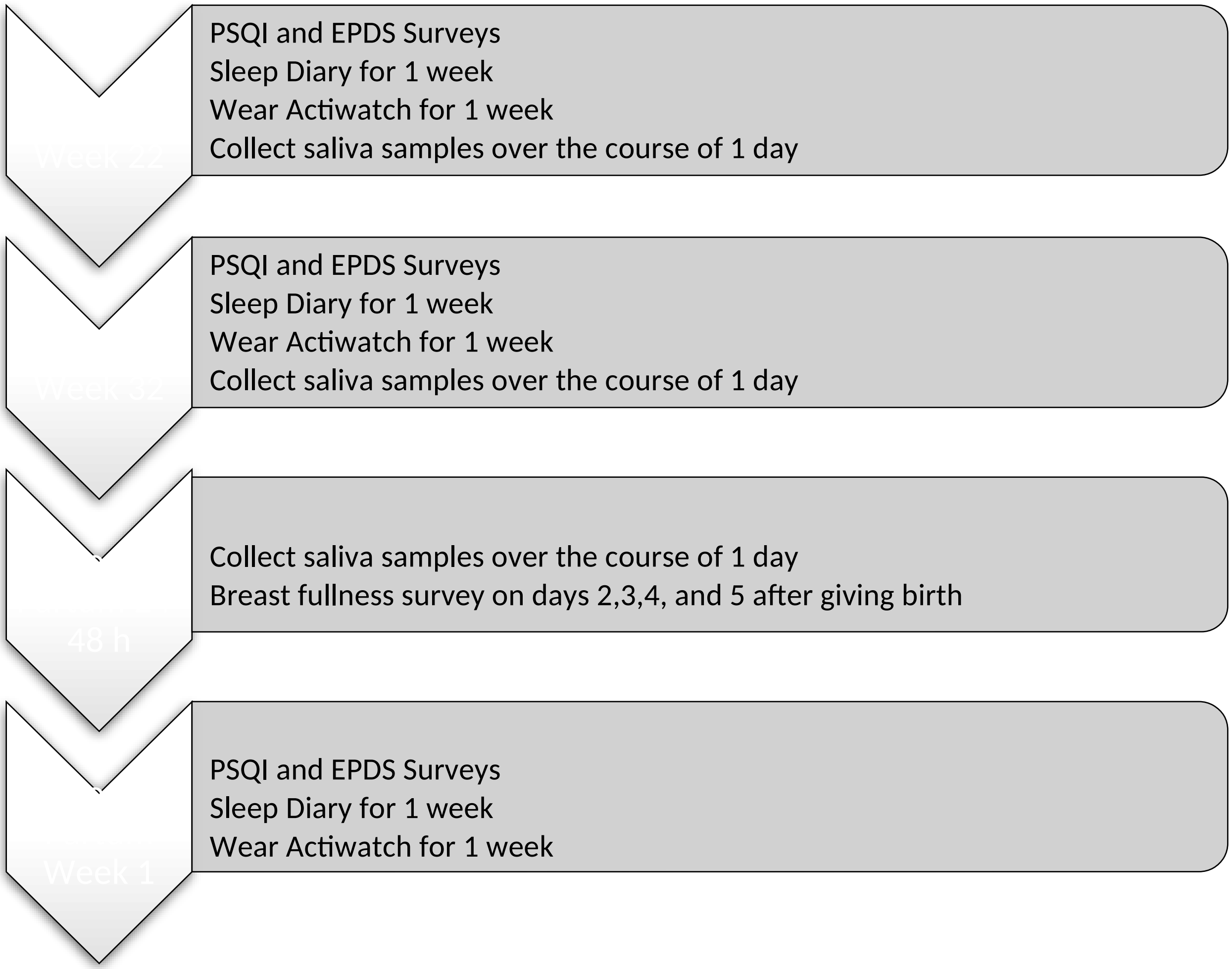
Week 22
PSQI and EPDS Surveys
Sleep Diary for 1 week
Wear Actiwatch for 1 week
Collect saliva samples over the course of 1 day
Week 32
PSQI and EPDS Surveys
Sleep Diary for 1 week
Wear Actiwatch for 1 week
Collect saliva samples over the course of 1 day
48 h
Collect saliva samples over the course of 1 day
Breast fullness survey on days 2,3,4, and 5 after giving birth
Week 1
PSQI and EPDS Surveys
Sleep Diary for 1 week
Wear Actiwatch for 1 week
