# Supplementary material for: Pregnancy rest-activity patterns are related to salivary cortisol rhythms and maternal-fetal health indicators in women from a disadvantaged population
Source: PLoS One. 2020 Mar 3;15(3):e0229567. doi: 10.1371/journal.pone.0229567 (PMC7053712; doi:10.1371/journal.pone.0229567)
Supplement: Supplementary file 3 [file pone.0229567.s002.docx]

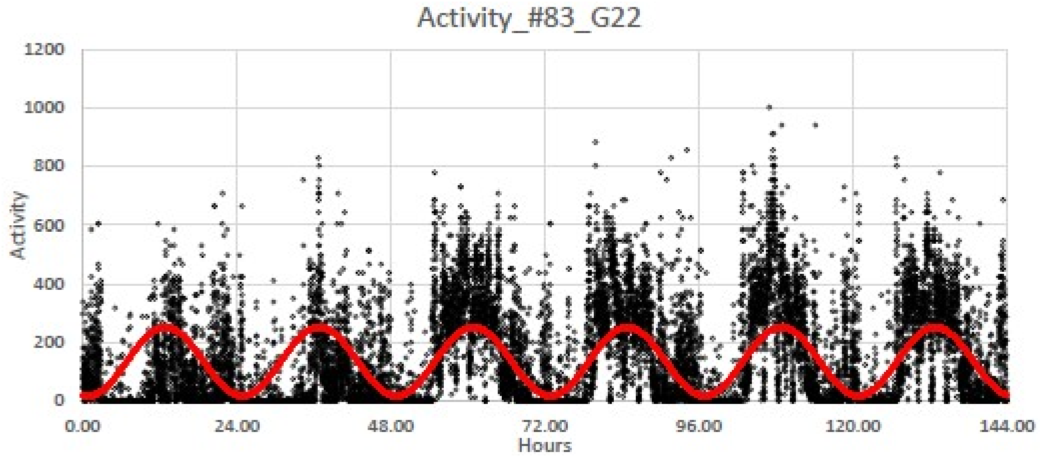
Activity_#83_G22
1200
1000
800
600
400
200
0
Activity
0.00
24.00
48.00
72.00
96.00
120.00
144.00
Hours

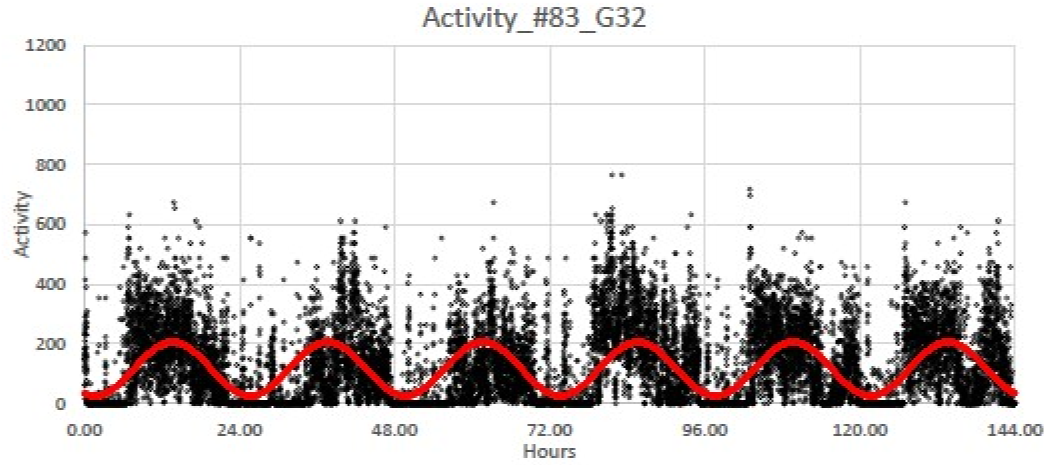
Activity_#83_G32
1200
1000
800
600
400
200
0
Activity
0.00
24.00
48.00
72.00
96.00
120.00
144.00
Hours

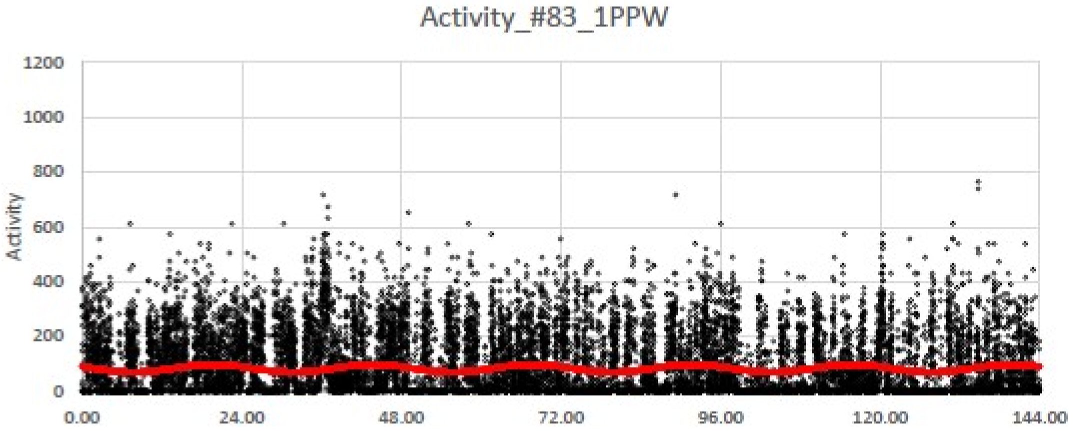
Activity_#83_1PPW
1200
1000
800
600
400
200
0
Activity
0.00
24.00
48.00
72.00
96.00
120.00
144.00

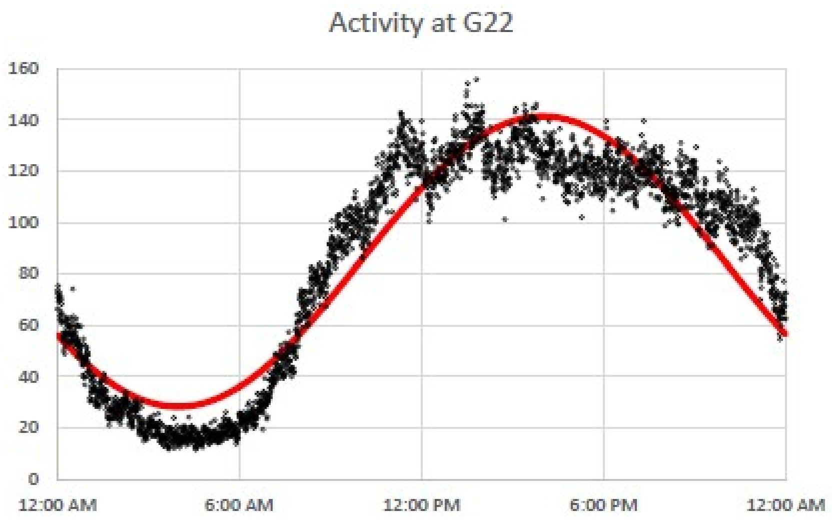

Activity at G22
160
140
120
100
80
60
40
20
0
12:00 AM
6:00 AM
12:00 PM
6:00 PM
12:00 AM

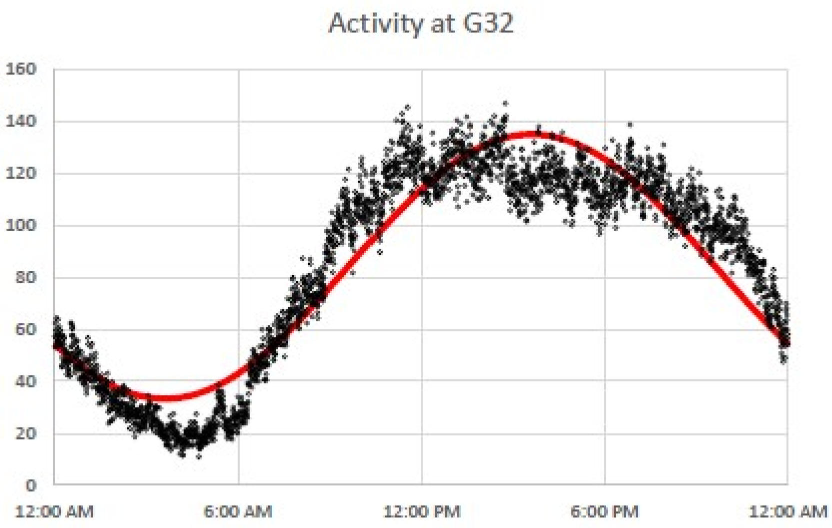

Activity at G32
160
140
120
100
80
60
40
20
0
12:00 AM
6:00 AM
12:00 PM
6:00 PM
12:00 AM

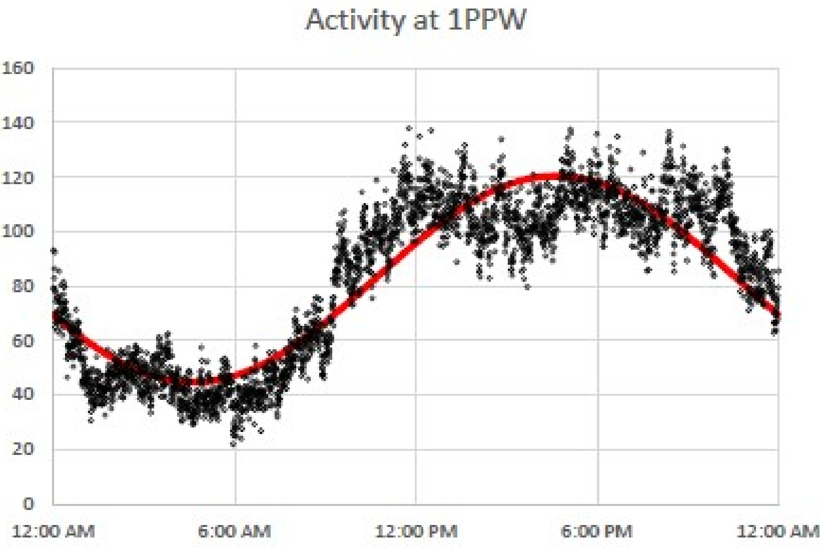

Activity at 1PPW
160
140
120
100
80
60
40
20
0
12:00 AM
6:00 AM
12:00 PM
6:00 PM
12:00 AM
